# Supplementary material for: Empirical methods for controlling false positives and estimating confidence in ChIP-Seq peaks
Source: BMC Bioinformatics. 2008 Dec 5;9:523. doi: 10.1186/1471-2105-9-523 (PMC2628906; doi:10.1186/1471-2105-9-523)
Supplement: Additional File 1 — A variety of html documents from the USeq web site detailing the available applications, their best usage, output file type descriptions, command line menus, etc. [file 1471-2105-9-523-S1.zip › USeqUserGuides/JavaDoc.zip!/JavaDoc/util/gen/Swing.html]

Swing


|  |  |  |
| --- | --- | --- |
| **Overview** \| **Package** \| **Class** \| **Use** \| **Tree** \| **Deprecated** \| **Index** \| **Help** | |  |
| **PREV CLASS**   **NEXT CLASS** | **FRAMES**    **NO FRAMES**     **All Classes** |  |
| SUMMARY: NESTED \| FIELD \| CONSTR \| METHOD | DETAIL: FIELD \| CONSTR \| METHOD |  |


---


## util.gen Class Swing

```
java.lang.Object
  util.gen.Swing
```

---

``` public class Swing extends java.lang.Object ```

Methods related to Swing apps.

---

**Constructor Summary**

`Swing()`


| **Method Summary** | |
| --- | --- |
| `static void` | `drawJLabel(java.awt.Graphics2D g2, javax.swing.JLabel label, float tuningFraction)`             Draws a JLabel on a Graphics2D. |
| `static java.awt.RenderingHints` | `fetchRenderingHints()`             Map of hints for high quality/ slow rendering |
| `static javax.swing.JButton` | `makeButton(java.lang.String text, int size, java.awt.event.ActionListener action, javax.swing.JPanel panel)` |
| `static double` | `makeDoubleInputDialog(java.lang.String title, java.lang.String comment, double suggestion)` |
| `static double` | `makeDoublePercentInputDialog(java.lang.String title, java.lang.String comment, double suggestion)` |
| `static javax.swing.JTextField` | `makeFieldSimple(javax.swing.JPanel panel, java.lang.String text)` |
| `static int` | `makeIntInputDialog(java.lang.String title, java.lang.String comment, int suggestion)` |
| `static javax.swing.JLabel` | `makeLabel(javax.swing.JPanel panel, java.lang.String text, int size, int thickness)` |
| `static int[]` | `setButton(javax.swing.JButton but, int x, int y)` |
| `static int[]` | `setField(javax.swing.JTextField field, int x, int y, int width)` |
| `static int[]` | `setLabel(javax.swing.JLabel lab, int x, int y)` |
| `static void` | `showFileChooserDialogBox(javax.swing.JTextField fieldRef, javax.swing.JFrame frame, javax.swing.JFileChooser chooser)` |
| `static void` | `throwError(javax.swing.JFrame frame, java.lang.String text)` |
| `static void` | `throwWarning(javax.swing.JFrame frame, javax.swing.JTextField field, java.lang.String text)` |

**Methods inherited from class java.lang.Object**

`equals, getClass, hashCode, notify, notifyAll, toString, wait, wait, wait`

**Constructor Detail**

### Swing

```
public Swing()
```


**Method Detail**

### fetchRenderingHints

```
public static java.awt.RenderingHints fetchRenderingHints()
```

:   Map of hints for high quality/ slow rendering

---


### drawJLabel

```
public static void drawJLabel(java.awt.Graphics2D g2,
                              javax.swing.JLabel label,
                              float tuningFraction)
```

:   Draws a JLabel on a Graphics2D. Set the tuningFraction to 1.05 for standard drawing or adjust to modify the y displacement.
    For some blasted reason, JLabels don't draw for buffered images when the layout manager is null. Thus this work around.

---


### makeDoublePercentInputDialog

```
public static double makeDoublePercentInputDialog(java.lang.String title,
                                                  java.lang.String comment,
                                                  double suggestion)
```

---


### makeDoubleInputDialog

```
public static double makeDoubleInputDialog(java.lang.String title,
                                           java.lang.String comment,
                                           double suggestion)
```

---


### makeIntInputDialog

```
public static int makeIntInputDialog(java.lang.String title,
                                     java.lang.String comment,
                                     int suggestion)
```

---


### throwWarning

```
public static void throwWarning(javax.swing.JFrame frame,
                                javax.swing.JTextField field,
                                java.lang.String text)
```

---


### throwError

```
public static void throwError(javax.swing.JFrame frame,
                              java.lang.String text)
```

---


### setButton

```
public static int[] setButton(javax.swing.JButton but,
                              int x,
                              int y)
```

---


### makeButton

```
public static javax.swing.JButton makeButton(java.lang.String text,
                                             int size,
                                             java.awt.event.ActionListener action,
                                             javax.swing.JPanel panel)
```

---


### showFileChooserDialogBox

```
public static void showFileChooserDialogBox(javax.swing.JTextField fieldRef,
                                            javax.swing.JFrame frame,
                                            javax.swing.JFileChooser chooser)
```

---


### makeFieldSimple

```
public static javax.swing.JTextField makeFieldSimple(javax.swing.JPanel panel,
                                                     java.lang.String text)
```

---


### makeLabel

```
public static javax.swing.JLabel makeLabel(javax.swing.JPanel panel,
                                           java.lang.String text,
                                           int size,
                                           int thickness)
```

---


### setField

```
public static int[] setField(javax.swing.JTextField field,
                             int x,
                             int y,
                             int width)
```

---


### setLabel

```
public static int[] setLabel(javax.swing.JLabel lab,
                             int x,
                             int y)
```


---


|  |  |  |
| --- | --- | --- |
| **Overview** \| **Package** \| **Class** \| **Use** \| **Tree** \| **Deprecated** \| **Index** \| **Help** | |  |
| **PREV CLASS**   **NEXT CLASS** | **FRAMES**    **NO FRAMES**     **All Classes** |  |
| SUMMARY: NESTED \| FIELD \| CONSTR \| METHOD | DETAIL: FIELD \| CONSTR \| METHOD |  |


---
